# MPLEx: a Robust and Universal Protocol for Single-Sample Integrative Proteomic, Metabolomic, and Lipidomic Analyses

**DOI:** 10.1128/mSystems.00043-16

**Published:** 2016-05-10

**Authors:** Ernesto S. Nakayasu, Carrie D. Nicora, Amy C. Sims, Kristin E. Burnum-Johnson, Young-Mo Kim, Jennifer E. Kyle, Melissa M. Matzke, Anil K. Shukla, Rosalie K. Chu, Athena A. Schepmoes, Jon M. Jacobs, Ralph S. Baric, Bobbie-Jo Webb-Robertson, Richard D. Smith, Thomas O. Metz

**Affiliations:** a Earth & Biological Sciences Directorate, Pacific Northwest National Laboratory, Richland, Washington, USA; b Department of Epidemiology, University of North Carolina at Chapel Hill, Chapel Hill, North Carolina, USA; c Department of Microbiology and Immunology, University of North Carolina at Chapel Hill, Chapel Hill, North Carolina, USA; d National Security Directorate, Pacific Northwest National Laboratory, Richland, Washington, USA; Mayo Clinic

**Keywords:** metabolomics, multi-omics analysis, lipidomics, proteomics, sample preparation, MERS-CoV

## Abstract

Integrative multi-omics analyses can empower more effective investigation and complete understanding of complex biological systems. Despite recent advances in a range of omics analyses, multi-omic measurements of the same sample are still challenging and current methods have not been well evaluated in terms of reproducibility and broad applicability. Here we adapted a solvent-based method, widely applied for extracting lipids and metabolites, to add proteomics to mass spectrometry-based multi-omics measurements. The metabolite, protein, and lipid extraction (MPLEx) protocol proved to be robust and applicable to a diverse set of sample types, including cell cultures, microbial communities, and tissues. To illustrate the utility of this protocol, an integrative multi-omics analysis was performed using a lung epithelial cell line infected with Middle East respiratory syndrome coronavirus, which showed the impact of this virus on the host glycolytic pathway and also suggested a role for lipids during infection. The MPLEx method is a simple, fast, and robust protocol that can be applied for integrative multi-omic measurements from diverse sample types (e.g., environmental, *in vitro*, and clinical).

**IMPORTANCE** In systems biology studies, the integration of multiple omics measurements (i.e., genomics, transcriptomics, proteomics, metabolomics, and lipidomics) has been shown to provide a more complete and informative view of biological pathways. Thus, the prospect of extracting different types of molecules (e.g., DNAs, RNAs, proteins, and metabolites) and performing multiple omics measurements on single samples is very attractive, but such studies are challenging due to the fact that the extraction conditions differ according to the molecule type. Here, we adapted an organic solvent-based extraction method that demonstrated broad applicability and robustness, which enabled comprehensive proteomics, metabolomics, and lipidomics analyses from the same sample.

**Author Video**: An author video summary of this article is available.

## Abstract (precis)

In systems biology studies, the integration of multiple omics measurements (i.e., genomics, transcriptomics, proteomics, metabolomics, and lipidomics) has been shown to provide a more complete and informative view of biological pathways. Thus, the prospect of extracting different types of molecules (e.g., DNAs, RNAs, proteins, and metabolites) and performing multiple omics measurements on single samples is very attractive, but such studies are challenging due to the fact that the extraction conditions differ according to the molecule type. Here, we adapted an organic solvent-based extraction method that demonstrated broad applicability and robustness, which enabled comprehensive proteomics, metabolomics, and lipidomics analyses from the same sample.

## INTRODUCTION

Multi-omic measurements and the integration of the resulting information can transform our understanding of complex biological systems (1–4). Multiple layers of information (DNAs, RNAs, proteins, metabolites, and lipids) can provide key insights regarding regulatory networks that are often overlooked using a single type of measurement (e.g., only proteomics or metabolomics). For instance, changes in levels of a given metabolite can be measured by metabolomics, which can result from the regulation of either its biosynthetic or degradation pathways. However, also measuring the levels of enzymes of each pathway using proteomics can reveal which mechanism is being regulated. Further, measurements of the enzyme RNA levels can also provide key information on whether the regulation occurs at the transcriptional or posttranscriptional level. For example, Bordbar et al. built a metabolic network model based on available genomic sequences to study macrophage activation and subsequently used transcriptomics, proteomics, and metabolomics information to further refine the model, which led to a better understanding of the impact of metabolism during an inflammatory response (1).

In the context of multi-omics analyses, being able to perform multiple measurements on the same sample can also decrease experimental variation. Additionally, this approach can be very useful when samples are difficult to obtain, i.e., for some environmental and patient samples (e.g., biopsy specimens) and for samples from high-biosafety-level laboratories, where working conditions are not optimal and are otherwise rigorously controlled. In addition, limited volumes or amounts of samples may preclude splitting them prior to performing parallel extractions and sample processing. Recent studies have evaluated the use of variations of chloroform/methanol extraction methods to isolate proteins, metabolites, and lipids or to sequentially extract DNA, RNA, proteins, metabolites, and lipids, sometimes with the use of different commercial kits, and all from the same sample (5–9). While the use of chloroform/methanol mixtures is well established for metabolomics and lipidomics sample preparation (we routinely use such a protocol in our laboratory), the reproducibility of proteomics, transcriptomics, and genomics measurements and their applicability for a diverse range of samples require further investigation. Indeed, we have found only a single report of an evaluation of the reproducibility of extraction of RNA and protein and of the reproducibility of the resulting proteomics data from a single sample type; Weckwerth et al. found that RNA and protein that were extracted from *Arabidopsis thaliana* had coefficients of variation (CVs) of 30% and 17%, respectively, when using a multi-omic extraction protocol based on the use of chloroform/methanol (7). Targeted quantification of peptides mapping to 22 proteins showed CVs of 17% on average. Recent analysis of the material obtained using different kits for multiple extractions showed reduced yields and/or quality of the end products (10). This could have been due to the fact that optimum buffers and solutions differ for extracting DNA, RNA, proteins, or metabolites and that longer extraction protocols may lead to material degradation.

Methods employing organic solvent extractions, such as the combination of chloroform, methanol, and water, have been widely used for extracting lipids and other metabolites (11, 12). In this procedure, a chloroform and methanol solution is added to samples resuspended in water or aqueous buffer, or directly to samples that have sufficient water content, so as to induce the formation of two solvent layers—an upper aqueous phase, containing hydrophilic metabolites, and a lower organic phase, containing lipids and other hydrophobic metabolites—while proteins precipitate in the interphase. Since organic solvent extraction is a simple and quick procedure, we reasoned, as others have (5–8), that it would prevent protein loss by degradation and make possible the simultaneous extraction of lipids, metabolites, and proteins for subsequent omics analyses. Furthermore, organic solvents can be easily removed by evaporation, minimizing the introduction of artifacts during sample preparation.

In this work, we sought to develop a robust protocol for simultaneous metabolite, protein, and lipid extraction (MPLEx) from the same samples for integrative multi-omic analyses. We based the protocol on a chloroform-methanol-water extraction method routinely used in our laboratory to simultaneously prepare metabolite and lipid extracts from the same sample. Others have demonstrated the reproducibility of the resulting metabolomics and lipidomics data in using variations of this protocol for select sample types (5, 7, 9, 13). To evaluate the broad applicability of expansion of this method for proteomics, we performed comprehensive proteomics analyses of the protein material extracted with the MPLEx procedure from a variety of samples, including a Gram-negative bacterium, an archaeon, an environmental microbial community, a plant leaf, a murine tissue, a human body fluid, and a cell line. We found that the proteome coverage for this diverse set of samples was very similar to that seen with matched control samples prepared in parallel using a standard proteomics sample preparation method, suggesting the broad applicability of the protocol. We then applied this methodology and integrated proteomic, lipidomic, and metabolomic analyses in the study of Middle East respiratory syndrome coronavirus (MERS-CoV) infections in a lung epithelial cell line, which showed the impact of viral infection on different host metabolic pathways.

## RESULTS AND DISCUSSION

### Impact of different metabolite extraction methods on proteomic analysis.

Integrative multi-omics analysis is a powerful approach to study complex biological responses and has gained popularity in recent years (1–3). In this context, the prospect of being able to perform multiple omics measurements on the same sample is very attractive but the method is still difficult to implement, likely due to the distinct optimal conditions for extracting different types of molecules. Aiming to develop a protocol for global multi-omic analyses of the same sample, we modified an extraction approach based on a chloroform-methanol-water solution to simultaneously obtain metabolite, protein, and lipid fractions. Since the protocol is well established and since we have applied it successfully for the analysis of lipids and other metabolites in several studies (14–19), we focused our efforts on determining if the method is applicable for global proteomic analysis and the associated quantification of relative amounts of proteins (i.e., the determination of fold increase or decrease in protein expression). We tested the MPLEx method with the Gram-negative bacterium *Shewanella oneidensis* by extracting its proteins, lipids, and metabolites (*n* = 5). As a comparison, we also performed extractions using 100% methanol (MeOH) or 100% acetonitrile (ACN) (*n* = 5 [each]), which are commonly used solvents for metabolomics extractions.

**FIG 1:**
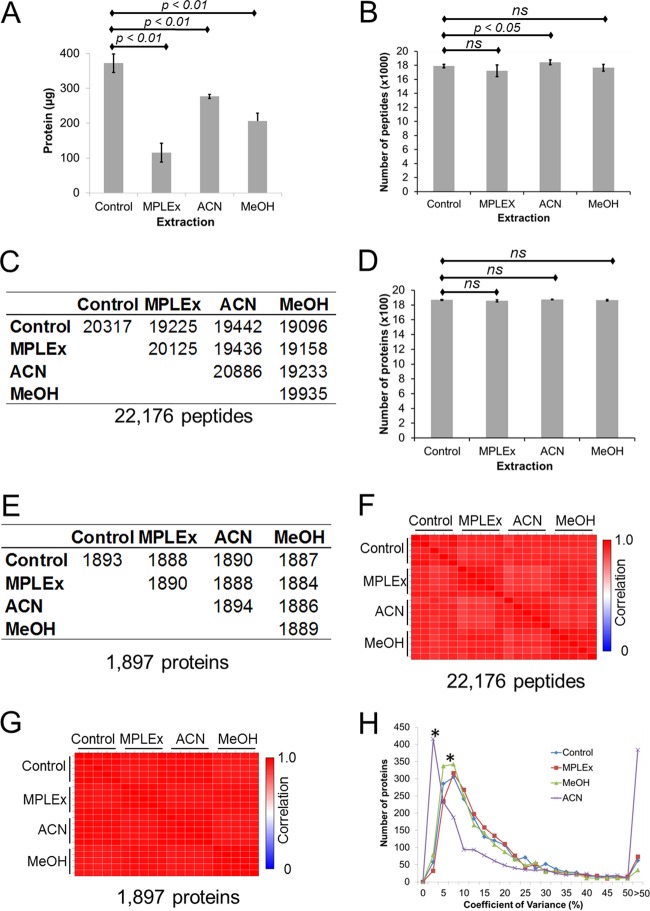
Extraction of *S. oneidensis* proteins with metabolite, protein, and lipid extraction (MPLEx), acetonitrile (ACN), and methanol (MeOH). A parallel sample was digested with trypsin without previous extraction (Control) as a control. (A) Protein recovery after extraction. (B) Numbers of identified peptides in different extractions. ns, not significant. (C) Matrix showing the numbers of overlapping peptides identified in samples extracted with different methods. In the matrix, the numbers of common peptides are indicated in the intersections between sample rows and columns. (D) Numbers of identified proteins in different extractions. (E) Matrix showing the numbers of overlapping proteins identified in samples extracted with different methods. (F) Correlation of peptide intensities of samples extracted with different methods. (G) Correlation of protein intensities of samples extracted with different methods. (H) Distribution of coefficients of variance across proteins identified in samples extracted with different methods. *, *P* ≤ 0.001 (compared to control sample).

We found that significantly reduced total protein fractions were recovered after extraction of metabolites and lipids by all three methods compared to control samples prepared using a standard protocol (Control) (Fig. 1A). These results are in agreement with previous data from the literature showing that some protein mass is lost during precipitation procedures (20). We then evaluated if these protein losses affected the ability to obtain useful proteomic data, since a method that can simultaneously extract multiple omics sources from the same sample would be extremely useful for systems biology experiments and subsequent integrated data analysis, as well as in cases where limited sample amounts are available (e.g., a survey of data from the National Cancer Institute showed that obtaining an adequate number of samples to conduct a study is a major difficulty facing researchers [21]). Thus, we investigated whether extraction with organic solvents would have a major impact on the coverage and the quantitative aspect of the associated proteomic analysis. To explore this issue, proteins extracted with MPLEx, ACN, and MeOH methods were digested in parallel with control samples, normalized by bicinchoninic acid (BCA) assay, and analyzed by liquid chromatography mass spectrometry (LC-MS) using the accurate mass & time (AMT) tag approach (22). The results of the proteomic analysis of samples extracted with different methods showed that the numbers of peptides detected in the MPLEx samples were very similar (no significant difference) to the numbers seen with controls (Fig. 1B). A significant increase in the levels of peptides was identified in samples extracted with ACN, but no significant differences between the control and MeOH extractions in the numbers of peptides were observed (Fig. 1B). The overlap of the numbers of peptides identified in samples extracted with all protocols was very high, as shown by a similarity matrix (Fig. 1C). The similarities between samples were even higher at the protein level (Fig. 1D and E). The similarity of the proteome coverage results obtained by the different extraction methods is remarkable, considering that much larger (up to 3-fold to 4-fold) differences are observed just by digesting proteins using different buffers, surfactants, or denaturing agents, even without any previous extraction (23, 24). Our results show that despite some protein mass losses, the choice of extraction protocol did not significantly affect the proteome coverage. The selective loss of a few proteins during the extraction procedure is expected and has been shown in a study carried out with human plasma samples only (20).

Another important feature for multi-omic analysis is that of being able to accurately identify differentially expressed or abundant molecules. In this context, if the extraction procedure affects the quality of the proteins, then it might increase the variance across different samples. Thus, we examined the correlation of the proteomics data between samples extracted with different organic solvents, and the results showed remarkable similarity at both peptide and protein levels (Fig. 1F and G). We then calculated the variance of protein measurements by comparing different extraction protocols. Indeed, no significant differences in the distributions of coefficients of variance (CV) were observed comparing MPLEx with controls, with the CVs of the great majority of the proteins smaller than 25%, with peaks of <10% (Fig. 1H). MeOH extraction led to CVs that were similar to but slightly smaller than those seen with the MPLEx and control samples (Fig. 1H). On the other hand, ACN extraction had a bimodal distribution, with very low and very high CVs (Fig. 1H), suggesting that some proteins are not reproducibly precipitated with this solvent. This phenomenon might be due to the fact that acetonitrile does not fully precipitate small proteins (25). Taken together, these results showed that MPLEx did not affect the proteome coverage or the results of quantitative analysis of the *S. oneidensis* samples.

### Performance of MPLEx in the analysis of different sample types.

**FIG 2:**
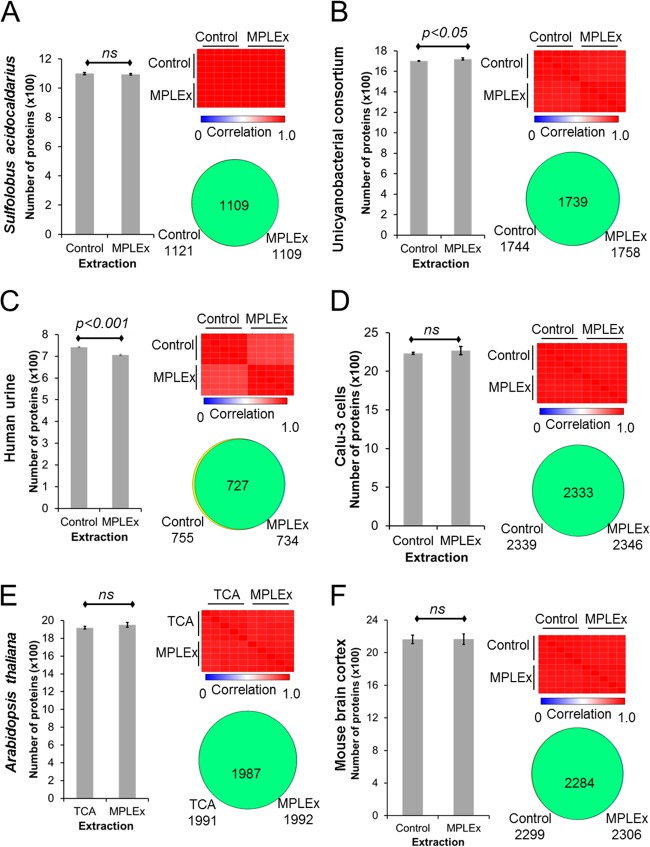
Proteomic coverage of diverse sets of samples. (A) The archaeon *S. acidocaldarius*. (B) Unicyanobacterial consortium. (C) Human urine. (D) Human lung epithelial cell line Calu-3. (E) *A. thaliana* plant leaves. (F) Mouse brain cortex. Each figure shows the number of identified proteins, correlation between replicates, and proteome coverage. Abbreviations: MPLEx, metabolite, protein, and lipid extraction; Control, no-extraction control; TCA, trichloroacetic acid extraction. All samples were prepared and measured in 5 replicates and analyzed by *t* test, assuming two tails and equal distributions.

To investigate whether the MPLEx protocol is robust and broadly applicable, we performed proteomic analyses of a very diverse set of samples that included the archaeon *Sulfolobus acidocaldarius*, a unicyanobacterial consortium (26), mouse brain cortex tissue, human urine, cells of the Calu-3 human lung epithelial cell line, and leaves from *Arabidopsis thaliana*. Whereas we compared MPLEx results to control results for most of these samples, the *A. thaliana* sample results were compared to results of extractions performed with saturated phenol or trichloroacetic acid (TCA), because plant leaves are rich in phenolic compounds that need to be removed and that otherwise would interfere with mass spectrometric analysis, and these alternative protocols have been shown to perform well in preparations of plant samples (27). As observed for *S. oneidensis*, the proteome coverage was very high at both the peptide (see Fig. S1 in the supplemental material) and protein (Fig. 2) levels across the diverse set of samples when using MPLEx and comparable to that obtained using the standard protein extraction protocol, although minor differences were detected for the unicyanobacterial consortium and human urine samples. In the case of *A. thaliana*, similar proteome coverage results were observed in samples extracted using either TCA or MPLEx (Fig. 2E; see also Fig. S1E). However, despite repeating the experiment twice, we had very limited success in extracting leaf proteins using the phenol protocol. In terms of quantitative measurements, similar correlations were observed across different samples by comparing MPLEx results to control or TCA extraction results at both the peptide and protein levels, although minor differences were observed in the results from the human urine samples (Fig. 2; see also Fig. S1). Overall, comparing MPLEx to control or TCA extraction, the levels of proteome coverage and correlation between samples were very similar (see Fig. S2), suggesting no qualitative losses.

10.1128/mSystems.00043-16.2 Figure S1  Proteomic coverage of diverse sets of samples. (a) The archaeon *S. acidocaldarius*. (b) Unicyanobacterial consortium. (c) Human urine. (d) Human lung epithelial cell line Calu-3. (e) *A. thaliana* plant leaves. Each figure shows the number of identified peptides, correlation between replicates, and proteome coverage. Abbreviations: MPLEx, metabolite, protein, and lipid extraction; noEx, no extraction; TCA, trichloroacetic acid extraction. All samples were prepared and measured in 5 replicates and analyzed by *t* test, assuming two tails and equal distributions. Download Figure S1, TIF file, 0.5 MB. Copyright © 2016 Nakayasu et al. 2016 Nakayasu et al. This content is distributed under the terms of the Creative Commons Attribution 4.0 International license.

10.1128/mSystems.00043-16.3 Figure S2  Coefficient of variance of proteomic measurements of a diverse set of samples. (a) The archaeon *Sulfolobus acidocaldarius*. (b) Unicyanobacterial consortium. (c) Human urine. (d) Human lung epithelial cell line Calu-3. (e) *A. thaliana* plant leaves. Abbreviations: MPLEx, metabolite, protein, and lipid extraction; noEx, no extraction; TCA, trichloroacetic acid extraction. All samples were prepared and measured in 5 replicates and analyzed by *t* test, assuming two tails and equal distributions. Download Figure S2, TIF file, 0.4 MB. Copyright © 2016 Nakayasu et al. 2016 Nakayasu et al. This content is distributed under the terms of the Creative Commons Attribution 4.0 International license.

The fact that the proteome coverage, correlation, and variability results of comparisons of samples using MPLEx are not different from those seen with the standard protocol indicates that the relative quantification of proteins, which is the type of quantification employed in the vast majority of proteomics studies, is not compromised. Nonetheless, we investigated any losses of specific proteins that could affect studies focusing on absolute quantification of protein copy numbers. Only 1.1% and 1.9% of the proteins in *Shewanella oneidensis* were shown to be significantly enriched and depleted by more than 2-fold, respectively (Table 1). The ACN extraction showed a smaller number of significantly enriched or depleted proteins, which was likely a consequence of the higher variability in the replicates observed using this solvent (Table 1). In contrast, the MeOH extraction showed much higher losses than MPLEx (Table 1). With the exception of the human urine sample, all samples had losses corresponding to less than 5% of the proteins (Table 1). To investigate possible causes of protein enrichment or depletion using MPLEx, several physical-chemical properties of the significantly enriched or depleted proteins were examined, including the number of proteins with transmembrane domains, molecular weight, length, hydrophobicity calculated by grand average of hydropathy (GRAVY), and isolectric point (pI). No pattern was consistently observed across the different samples for any of the tested physical-chemical properties, indicating that the small amount of enrichment or depletion of proteins induced by MPLEx is not based on such properties. Although these small differences in protein extraction results seen using MPLEx should be considered in proteomics studies employing absolute quantification, they likely do not introduce artifacts in the results, as these studies typically have very small (up to 15%) errors when stable isotope-labeled peptides are used as internal standards (28) and up to 2-fold to 3-fold variations in label-free analyses (29, 30).

**TABLE 1:** Comparative analysis of protein extractions^a^

Protein category and parameter	Value(s)								
	*Shewanella oneidensis*			*Arabiposis* *thaliana*	Calu-3 cells	Human urine	Mouse brain cortex	*Sulfolobus* *acidocaldarius*	Unicyanobacterial consortium
	MPLEx	ACN	MeOH	MPLEx	MPLEx	MPLEx	MPLEx	MPLEx	MPLEx
Enriched									
No. of proteins	20	9	26	111	42	130	55	37	78
% of total	1.1	0.5	1.4	5.6	1.8	17.1	2.4	3.3	4.4
Proteins with TMD^b^	5 (25%)	5 (55.6%)	13 (50%)	12 (10.8%)	4 (9.5%)	31 (23.8%)	12 (21.8%)	7 (18.9%)	13 (16.7%)
MW^c^	40,808 ± 26,150	44,400 ± 31,645	44,230 ± 21,553	34,789 ± 33,487	559,869 ± 35,360	57,950 ± 54,709	56,830 ± 62,457	35,533 ± 20,022	40,193 ± 25,185
Length (aa)	373 ± 241	406 ± 292	402 ± 198	313 ± 299	497 ± 311	525 ± 495	507 ± 557	309 ± 183	370 ± 232
GRAVY score^d^	−0.029 ± 0.434	−0.164 ± 0.588	0.096 ± 0.452	−0.195 ± 0.254	−0.255 ± 0.269	−0.375 ± 0.308	−0.286 ± 0.393	−0.056 ± 0.316	−0.090 ± 0.288
pI^e^	6.76 ± 1.71	7.06 ± 1.57	7.40 ± 1.66	6.30 ± 1.58	7.35 ± 1.67	6.75 ± 1.63	7.61 ± 1.80	7.67 ± 1.32	5.73 ± 1.42
Depleted									
No. of proteins	37	3	88	15	32	179	38	32	86
% of total	1.9	0.2	4.6	0.8	1.4	23.5	1.6	2.9	4.9
Proteins with TMD	2 (5.4%)	2 (66.7%)	4 (5%)	0	1 (3.1%)	60 (33.5%)	10 (26.3%)	1 (3.1%)	13 (15.1%)
MW	24,198 ± 16,914	39,929 ± 28,848	22,974 ± 14,236	68,158 ± 48,708	26,063 ± 24,477	67,746 ± 71,966	71,216 ± 96,637	23,857 ± 11,867	32,116 ± 27,627
Length (aa)	222 ± 158	365 ± 267	209 ± 129	617 ± 440	229 ± 210	617 ± 664	642 ± 856	212 ± 106	294 ± 251
GRAVY score	−0.104 ± 0.283	−0.057 ± 0.166	−0.182 ± 0.257	−0.316 ± 0.168	−0.719 ± 0.476	−0.258 ± 0.311	−0.301 ± 0.459	−0.197 ± 0.248	−0.301 ± 0.434
pI	6.14 ± 1.58	7.45 ± 1.43	5.85 ± 1.21	6.67 ± 1.49	7.30 ± 1.83	6.54 ± 1.39	6.68 ± 1.42	6.29 ± 1.14	5.61 ± 1.56
Total									
No. of proteins	1,898			1,996	2,351	762	2,320	1,121	1,763
Proteins with TMD	335 (17.6%)			190 (9.5%)	350 (14.9%)	216 (28.3%)	377 (16.2%)	69 (6.2%)	230 (13.0%)
MW	42,093 ± 28,556			47,463 ± 32,164	62,933 ± 63,256	62,704 ± 71,856	64,029 ± 67,423	36,211 ± 20,586	41,646 ± 29,689
Length (aa)	381 ± 261			430 ± 288	564 ± 565	571 ± 661	575 ± 605	322 ± 184	381 ± 270

a Values for differentially abundant proteins were determined by T and G tests, and the numbers of proteins with more the 2-fold enrichment or depletion are listed. aa, amino acids.

b TMD, transmembrane domain.

c MW, molecular weight.

d GRAVY, grand average of hydropathy.

e pI, isoelectric point.

Although protein oxidation is an important physiological posttranslational modification, it is also an artifact introduced during sample processing for proteomic analysis. Considering that there is more O_2_ dissolved in organic solvents than in water (31), it is reasonable to suspect that extraction performed with such solvents could increase the oxidation of peptides. Thus, the number of peptides containing oxidized methionine residues was counted in each sample, and an increase in methionine oxidation was observed only in the *S. acidocaldarius* sample extracted with the MPLEx protocol (see Fig. S3 in the supplemental material). However, the opposite trend was observed in *S. oneidensis*, mouse brain cortex, and unicyanobacterial consortium samples, and no difference was observed in the other samples (see Fig. S3). These results suggest that the oxidation of peptides is sample dependent and that it is not induced by MPLEx.

10.1128/mSystems.00043-16.4 Figure S3  Number of detected peptides containing oxidized methionine residues from the proteomic measurements of a diverse set of samples. (a) Gram-negative bacterium *Shewanella oneidensis*. (b) The archaeon *Sulfolobus acidocaldarius*. (c) Unicyanobacterial consortium. (d) Human urine. (e) Human lung epithelial cell line Calu-3. (f) *A. thaliana* plant leaves. Abbreviations: MPLEx, metabolite, protein, and lipid extraction; noEx, no extraction; TCA, trichloroacetic acid extraction. All samples were prepared and measured in 5 replicates and analyzed by *t* test, assuming two tails and equal distributions. Download Figure S3, TIF file, 0.4 MB. Copyright © 2016 Nakayasu et al. 2016 Nakayasu et al. This content is distributed under the terms of the Creative Commons Attribution 4.0 International license.

Taken together, our data show that MPLEx is a robust protocol and can be applied for a variety of sample types without compromising the proteome coverage or quantitative measurements or inducing oxidation artifacts.

### Application of MPLEx in multi-omics study of MERS-CoV infection in a lung epithelial cell line.

To illustrate an application for MPLEx and the value of multiple omics measurements obtained from the same sample, we applied the method to study MERS-CoV infection. We specifically chose MERS-CoV because it is a deadly emerging infectious agent with subsequent disease mortality rates of approximately 40% and because there are currently no effective drugs available for treatment (32). Since MERS-CoV is a newly emergent virus, information about the mechanism of virulence of the infection is very scarce in the literature and any new data would immensely contribute to a better understanding of the disease. In addition, experiments investigating MERS-CoV need to be performed in biosafety level 3 (BSL3) facilities, which require extensive safety and decontamination procedures. Thus, being able to analyze multiple omics from the same sample would significantly reduce the time of exposure risk of the researcher inside the biosafety facility.

**FIG 3:**
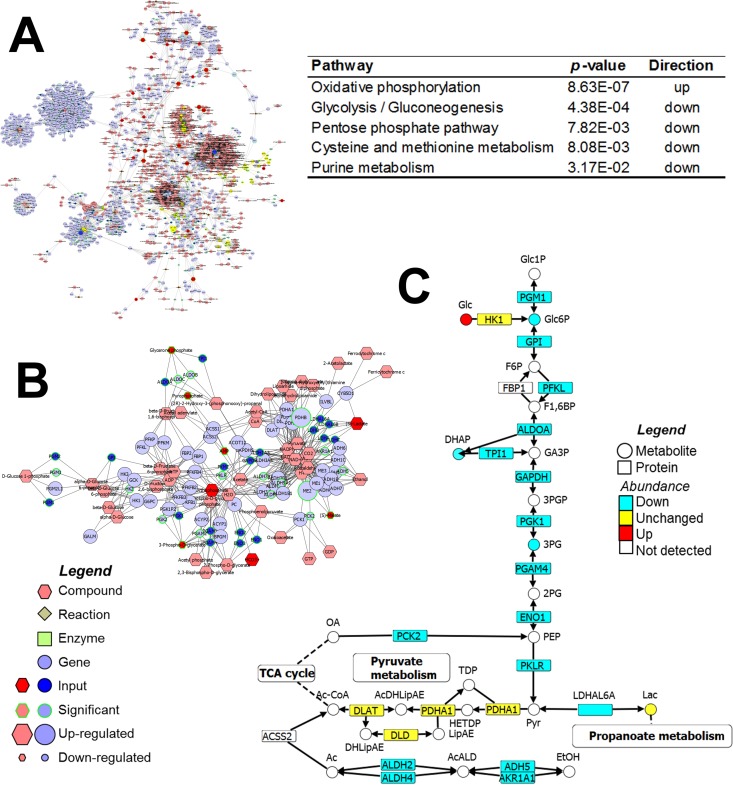
Integrative network of proteomics, metabolomics, and lipidomics of human lung epithelial Calu-3 cells infected with Middle East respiratory syndrome coronavirus (MERS-CoV). (A) Complete human metabolic network designed with Metscape and metabolic pathways enriched on differentially abundant proteins during viral infection. up, upregulation; down, downregulation. (B) Subnetwork of the glycolysis/gluconeogenesis pathway from Metscape analysis, which corresponds to the nodes highlighted in yellow in panel A. (C) Glycolysis/gluconeogenesis pathway manually curated using VANTED.

For this experiment, we used human lung epithelial Calu-3 cells, which we initially tested as described above and which showed good proteome coverage (Fig. 2D). Nine replicates of cell cultures were infected for 18 h with MERS-CoV, while 3 replicates were left uninfected as mock controls. Samples were subjected to MPLEx and submitted for global proteomic, metabolomic, and lipidomic analyses. In total, 2,670 proteins, 51 metabolites, and 236 lipid species were identified and quantified (see Tables S1 to S4 in the supplemental material). Data from all three global measurements were then integrated using the Metscape plugin of Cytoscape (Fig. 3A) (33, 34). We also performed a function-enrichment analysis based on the KEGG database using the LRpath tool (35) and combined this information into Metscape. The LRpath analysis showed that 25 pathways were significantly enriched in differentially abundant proteins (see Table S5) and that 5 of the pathways were from the central metabolism of the cell (Fig. 3A). From these pathways, we chose the glycolysis and gluconeogenesis pathways due to their complexity and the fact that these two pathways share most of the metabolites and enzymes therein. Being able to determine which of these pathways is affected more during infection would result in valuable information for better understanding the disease. In Fig. 3A, the nodes highlighted in yellow represent the glycolysis/gluconeogenesis pathway, which was separated into a subnetwork in Fig. 3B for a better visualization. This pathway showed several proteins that were downregulated during MERS-CoV infection, which are represented in Metscape by the small nodes (Fig. 3B). This pathway was then manually curated and visualized using the VANTED tool (36) (Fig. 3C), showing quantitatively that almost all proteins in the glycolysis/gluconeogenesis pathway were reduced in abundance during the infection with MERS-CoV (Fig. 3C). Although limited numbers of metabolites from the glycolysis/gluconeogenesis pathways were detected, the reduced levels of glucose 6-phosphate (G6P), dihydroxyacetone phosphate (DHAP), and 3-phospho-d-glycerate (3PG) further support the idea of a decrease in activity of this central pathway (Fig. 3C). Since glycolysis and gluconeogenesis share the same enzymes, proteomics alone is insufficient to determine exactly which process is affected. However, results from the addition of metabolomics, specifically, the observation that the initial substrate, glucose (Glc), had accumulated, indicated that glycolysis was more likely than gluconeogenesis to have been affected by the viral infection (Fig. 3C). To conclude, the proteomics analysis by itself would show differences only in the abundances of the enzymes from the glycolysis/gluconeogenesis pathway, but the addition of metabolite measurements helps confirm that the pathway activity is reduced and which direction is the more affected, clearly illustrating the advantage of integrating multi-omic measurements for studying specific metabolic pathways.

10.1128/mSystems.00043-16.5 Table S1  Global proteomic analysis of MERS-CoV-infected Calu-3 cells. Download Table S1, XLSX file, 0.9 MB. Copyright © 2016 Nakayasu et al. 2016 Nakayasu et al. This content is distributed under the terms of the Creative Commons Attribution 4.0 International license.

10.1128/mSystems.00043-16.6 Table S2  Metabolomics analysis of MERS-CoV-infected Calu-3 cells. Download Table S2, XLSX file, 0.02 MB. Copyright © 2016 Nakayasu et al. 2016 Nakayasu et al. This content is distributed under the terms of the Creative Commons Attribution 4.0 International license.

10.1128/mSystems.00043-16.7 Table S3  Lipidomics analysis of MERS-CoV-infected Calu-3 cells in positive ion mode. Download Table S3, XLSX file, 0.02 MB. Copyright © 2016 Nakayasu et al. 2016 Nakayasu et al. This content is distributed under the terms of the Creative Commons Attribution 4.0 International license.

10.1128/mSystems.00043-16.8 Table S4  Lipidomics analysis of MERS-CoV-infected Calu-3 cells in negative ion mode. Download Table S4, XLSX file, 0.02 MB. Copyright © 2016 Nakayasu et al. 2016 Nakayasu et al. This content is distributed under the terms of the Creative Commons Attribution 4.0 International license.

10.1128/mSystems.00043-16.9 Table S5  Function-enrichment analysis of proteins differentially abundant in MERS-CoV-infected Calu-3 cells. Download Table S5, XLSX file, 0.01 MB. Copyright © 2016 Nakayasu et al. 2016 Nakayasu et al. This content is distributed under the terms of the Creative Commons Attribution 4.0 International license.

10.1128/mSystems.00043-16.10 Table S6  Sequence databases, tools, and parameters used for peptide identification. Download Table S6, DOCX file, 0.01 MB. Copyright © 2016 Nakayasu et al. 2016 Nakayasu et al. This content is distributed under the terms of the Creative Commons Attribution 4.0 International license.

### MPLEx reveals global changes in lipid profiles induced by MERS-CoV infection.

**FIG 4:**
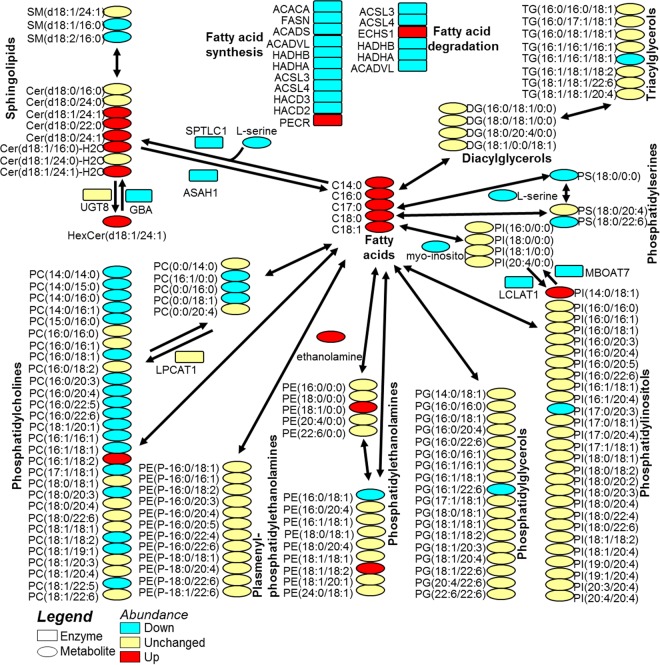
Lipid metabolic network integrating proteomics, metabolomics, and lipidomics of human lung epithelial Calu-3 cells infected with Middle East respiratory syndrome coronavirus (MERS-CoV).

To further demonstrate the utility of multi-omic analyses facilitated by the MPLEx protocol, we investigated MERS-CoV-stimulated changes in the Calu-3 lipidome by integrating the measurements of sphingolipids and glycerophospholipids from the lipidomics analysis, free fatty acids from the metabolomics analysis, and enzymes from the proteomic analysis using the VANTED tool (Fig. 4). Increases in the levels of all 5 detected fatty acid species were observed in MERS-CoV-infected cells compared to mock controls (Fig. 4). The increases in fatty acid levels appear unrelated to lipid synthesis itself, since almost all the enzymes of the synthesis pathway are downregulated with infection (Fig. 4). Conversely, the decrease in levels of enzymes in the fatty acid degradation pathway might be contributing to the accumulation of fatty acids (Fig. 4). In addition, degradation of phosphatidylcholines (PC), *lyso*-PC, phosphatidylserines (PS), and *lyso*-PS by phospholipases might also have been contributing to the accumulation of fatty acids during infection (Fig. 4). Although the responsible phospholipase was not detected in the proteomic analysis, it seems to be specific to PC and PS, since other classes of glycerophospholipids and glycerolipids remained mostly unchanged during infection (Fig. 4).

More-extensive changes in abundance were observed in members of sphingolipid classes than in phospholipids. The abundance of hexosylceramide increased during MERS-CoV infection, seemingly due to a decrease in the levels of its degradation enzyme glucosylceramidase (GBA) (Fig. 4). An increase of ceramide levels was also detected during infection which did not appear to be related to synthesis, since the abundance of serine palmitoyltransferase (SPTLC1), the enzyme that catalyzes the first step of ceramide synthesis by condensing serine and palmitate into 3-ketosphinganine, was decreased (Fig. 4). The accumulation of ceramides was most likely due to the degradation of sphingomyelin in combination with a decrease in levels of the ceramidase (ASAH1) (Fig. 4). Sphingolipids have been reported to play an integral role in viral uptake, replication, maturation, and budding during viral infection. Membrane domains enriched with ceramides have been proposed to facilitate the entry of enveloped viruses into host cells by changing the membrane fluidity and enhancing vesicular fusion (37). Ceramides are also known to trigger apoptosis and death of the host cells (38, 39). Indeed, apoptosis has already been reported in bronchial epithelial cells infected with MERS-CoV (40), but its relationship with the increased levels of ceramides still needs to be further investigated.

Overall, the lipid metabolic network built by integrating multi-omics measurements shows a much more complete and likely more accurate view of the lipid landscape compared to lipidomics alone and provides more insights concerning the mechanism of lipid regulation.

### Concluding remarks.

Integration of multi-omics measurements has been consolidated as a technique for studying complex biological systems (1–3). Thus, methods that enable multiple omics measurements on the same sample are not only attractive but the only choice in cases of samples with limited availability. In this context, the MPLEx method can be an excellent alternative since it has been shown to be robust and applicable for a variety of samples ranging from bacterial cells to environmental samples to animal tissue. It is worth noting that, in addition to metabolomics, proteomics, and lipidomics, it is very likely that MPLEx can be used for the analysis of posttranslational modifications. Indeed, a preliminary unpublished phosphoproteomic analysis using MPLEx led to the identification of several thousand phosphopeptides, although more careful analysis is required to determine if there are losses in this process. To conclude, we demonstrate the utility of multi-omics integration using MPLEx to study a lung epithelial cell line infected with MERS-CoV, which showed major differences in central carbon and lipid metabolism during infection.

## MATERIALS AND METHODS

### Samples.

For this study, we chose a variety of sample types: plant leaves from *Arapdopsis thaliana*, human urine as an example body fluid, the Gram-negative bacterium *Shewanella oneidensis*, the cultured tissue cell line Calu-3, a unicyanobacterial consortium isolated from Hot Lake, WA, USA (26), mouse brain cortex tissue, and the archaeon *Sulfolobus acidocaldarius* strain DSM 639. Calu-3 cell infection with MERS-CoV was performed as described in Text S1 in the supplemental material. *S. oneidensis*, the unicyanobacterial consortium, and *S. acidocaldarius* cells were lysed by bead beating in a Bullet Blender (Next Advance, Averill Park, NY) with 0.1-mm-diameter zirconia beads at speed 8 for 3 min at 4°C, and the lysate was spun into a Falcon tube at 2,000 × *g* for 10 min at 4°C. Additional lysis was done via pressure cycling technology (PCT) using a Barocycler (Pressure BioSciences Inc., South Easton, MA). The suspended cells were subjected to 20 s of high pressure at 35,000 lb/in^2^ followed by 10 s of ambient pressure for 10 cycles. *A. thaliana* leaves were frozen with liquid nitrogen and mechanically disrupted on a mortar with a pestle. Mouse brain cortex tissue was homogenized in ice-cold Nanopure H_2_O at full speed with a hand-held Omni tool and a disposable probe (Omni, Kennesaw, GA) for 30 s, allowed to cool, and homogenized again.

10.1128/mSystems.00043-16.1 Text S1  Supplemental materials and methods. Download Text S1, DOCX file, 0.04 MB. Copyright © 2016 Nakayasu et al. 2016 Nakayasu et al. This content is distributed under the terms of the Creative Commons Attribution 4.0 International license.

### Extraction methods.

Each sample was processed in 5 replicates using the following protocols.

### (i) Metabolite, protein, and lipid extraction (MPLEx).

The extraction procedure was adapted from the method of Folch et al. (41) by keeping the same final solvent proportions; however, the monophasic extraction step was not performed, as water was initially added to the sample along with the chloroform and methanol to simultaneously extract and partition molecules into the three different phases. Cell pellets or lysates were resuspended in water, and 5 volumes of cold (−20°C) chloroform-methanol (2:1 [vol/vol]) solution was added to the samples. Samples were incubated for 5 min on ice, subjected to vortex mixing for 1 min, and centrifuged at 12,000 rpm for 10 min at 4°C. For the samples for which metabolomics and lipidomics analyses were performed, the upper aqueous phase and bottom organic phase, containing hydrophilic metabolites and lipids, respectively, were collected in glass autosampler vials. The interphases, containing proteins, were washed by adding 1 ml of cold (−20°C) methanol, vortex mixed for 1 min, and centrifuged at 12,000 rpm for 10 min at 4°C. The supernatants were discarded, and the resulting pellets were dried in a vacuum centrifuge for 5 min.

### (ii) Phenol extraction.

Powdered *A. thaliana* leaves were resuspended in 10 ml of phenol extraction buffer (0.5 M Tris-HCl [pH 7.5], containing 0.7 M sucrose, 0.1 M KCl, 50 mM EDTA, 2% [vol/vol] β-mercaptoethanol, and 1 mM phenylmethanesulfonylfluoride), and then 10 ml of phenol solution saturated with 10 mM Tris-HCl (pH 7.5) was added to each tube. Samples were shaken for 30 min at 4°C and centrifuged at 5,000 × *g* for 30 min at 4°C. The upper phenolic phase was collected into a fresh tube and washed twice by adding 10 ml of phenol extraction buffer, followed by centrifugation at 5,000 × *g* for 30 min at 4°C, and discarding of the lower phase. The upper phenolic phase was collected in a fresh tube, and 5 volumes of 0.1 M ammonium acetate in methanol was added. Samples were incubated overnight at −20°C and centrifuged at 5,000 × *g* for 30 min at 4°C. Protein pellets were then washed twice with 10 ml ice-cold methanol and twice with 10 ml ice-cold acetone by adding the solvent, centrifuging at 5,000 × *g* for 30 min at 4°C, and discarding of the supernatant. The resulting protein pellet was dried under a stream of N_2_.

### (iii) TCA extraction.

10 ml of freshly prepared ice-cold TCA-acetone extraction buffer (0.61 M trichloroacetic acid–90% acetone) was added to powdered *A. thaliana* leaves, and the mixture was incubated overnight at −20°C. Proteins were then precipitated by centrifuging for 30 min at 5,000 × *g* at 4°C, and the supernatant was discarded. The protein pellet was washed three times by adding 10 ml of ice-cold acetone, followed by centrifugation for 10 min at 5,000 × *g* at 4°C, and discarding of the supernatant. The resulting protein pellet was dried under a stream of N_2_.

### (iv) Acetonitrile extraction.

Lysates were resuspended in 4 volumes of ice-cold (−20°C) pure acetonitrile and incubated for 10 min at 4°C to precipitate the proteins. The samples were centrifuged for 10 min at 4°C at 12,000 × *g* to pellet the protein. The supernatant was removed, and the protein pellets were dried by evaporation before digesting with trypsin.

### (v) Methanol extraction.

The methanol extraction was performed with the exact same procedure as the acetonitrile extraction, with the difference that the organic solvent was replaced by methanol.

### Proteomic, lipidomic, and metabolomic analyses.

The detailed methodology of proteomic, lipidomic, and metabolomic analyses are provided in Text S1 in the supplemental material. For proteomic analysis, proteins were digested with trypsin into peptides and analyzed using the accurate mass & time (AMT) tag approach (22). Peptides were separated by nano-capillary liquid chromatography (nano-LC), and eluting peptides were directly analyzed using LTQ-Orbitrap Velos or Exactive mass spectrometers (Thermo Fisher Scientific). Peptides were identified by matching to the appropriate mass tag database, and the peak areas were extracted using VIPER (42). Matching results were filtered with Statistical Tools for AMT tag confidence and uniqueness probability scores (43). Lipids extracted from Calu-3 cells infected with MERS-coronavirus were analyzed by LC-tandem MS (LC-MS/MS) using an LTQ-Orbitrap Velos mass spectrometer as previously described (14). Then, raw data files were analyzed using LIQUID (lipid informed quantitation and identification) software developed in-house for semiautomated identification of lipid molecular species followed by manual validation of identified species. Polar metabolites extracted from Calu-3 cells infected with MERS-coronavirus were derivatized with N-methyl-N-(trimethylsilyl)trifluoroacetamide (MSTFA) and analyzed by gas chromatography-mass spectrometry (GC-MS) as described previously (16). The raw data files were processed using MetaboliteDetector (44) and manually validated.

### Comparative analysis of different extractions.

The proteomic analyses comparing the different extraction methods were performed by rolling up the intensity values of peptides into values corresponding to proteins using the R rollup function of Inferno RDN (formerly DAnTE) (45). Only proteins with two or more peptides that were unique were considered for further analysis. The intensity values were transformed to log_2_ values and submitted to standard paired *t* tests and G tests (46) (considering only proteins present in 0 or 1 of 5 replicates).

### Statistical analysis of MERS-CoV-infected cells.

For analyses of proteomics, lipidomics, and metabolomics data from the Calu-3 cells infected with MERS-CoV, the quantitative data profiles were evaluated for extreme outlier behavior (47). No outlier samples were observed in the metabolomics and lipidomics data; however, one proteomics replicate from the infected group showed extremely poor coverage and correlation, indicating an issue with the protein extraction. That one sample was removed from subsequent analyses. Further quality assessment of the proteomics data included evaluation of individual peptides to identify those with inadequate coverage for either statistical analyses or protein quantification (46). Metabolomic and lipidomic data were normalized via standard median centering, and proteomics data were normalized via median centering against a rank-invariant peptide subset identified to reduce bias (48). To allow evaluation of the proteomic data at the protein level, a signature-based protein quantitation methodology was employed (49). Finally, the protein, metabolite, and lipid data were evaluated for quantitative differences between the results of mock infection and MERS-CoV infection via a standard two-sample *t* test.

### Multi-omics data integration.

Accession numbers from proteomics data of the MERS-CoV-infected cells were converted into Entrez Gene identifiers (ID) and uploaded to LRpath for function-enrichment analysis (35). Then, expression values of metabolomics, lipidomics (both converted to KEGG compound IDs), and proteomics data were integrated using Metscape v. 3.1.1 (33) plugin of cytoscape v3.2.1 (34) along with the function-enrichment results from LRpath analysis. Specific pathways of interest were manually curated using VANTED v2.2.0 (36).

### Accession numbers.

All LC-MS/MS and GC-MS data files were deposited into the MassIVE repository (http://massive.ucsd.edu/) under accession numbers MSV000079410, MSV000079409, MSV000079408, MSV000079407, MSV000079406, MSV000079405, MSV000079404, MSV000079609, and MSV000079610.
