# Supplementary figures and images for: MPLEx: a Robust and Universal Protocol for Single-Sample Integrative Proteomic, Metabolomic, and Lipidomic Analyses
Source: mSystems. 2016 May 10;1(3):e00043-16. doi: 10.1128/mSystems.00043-16 (PMC5069757; doi:10.1128/mSystems.00043-16)

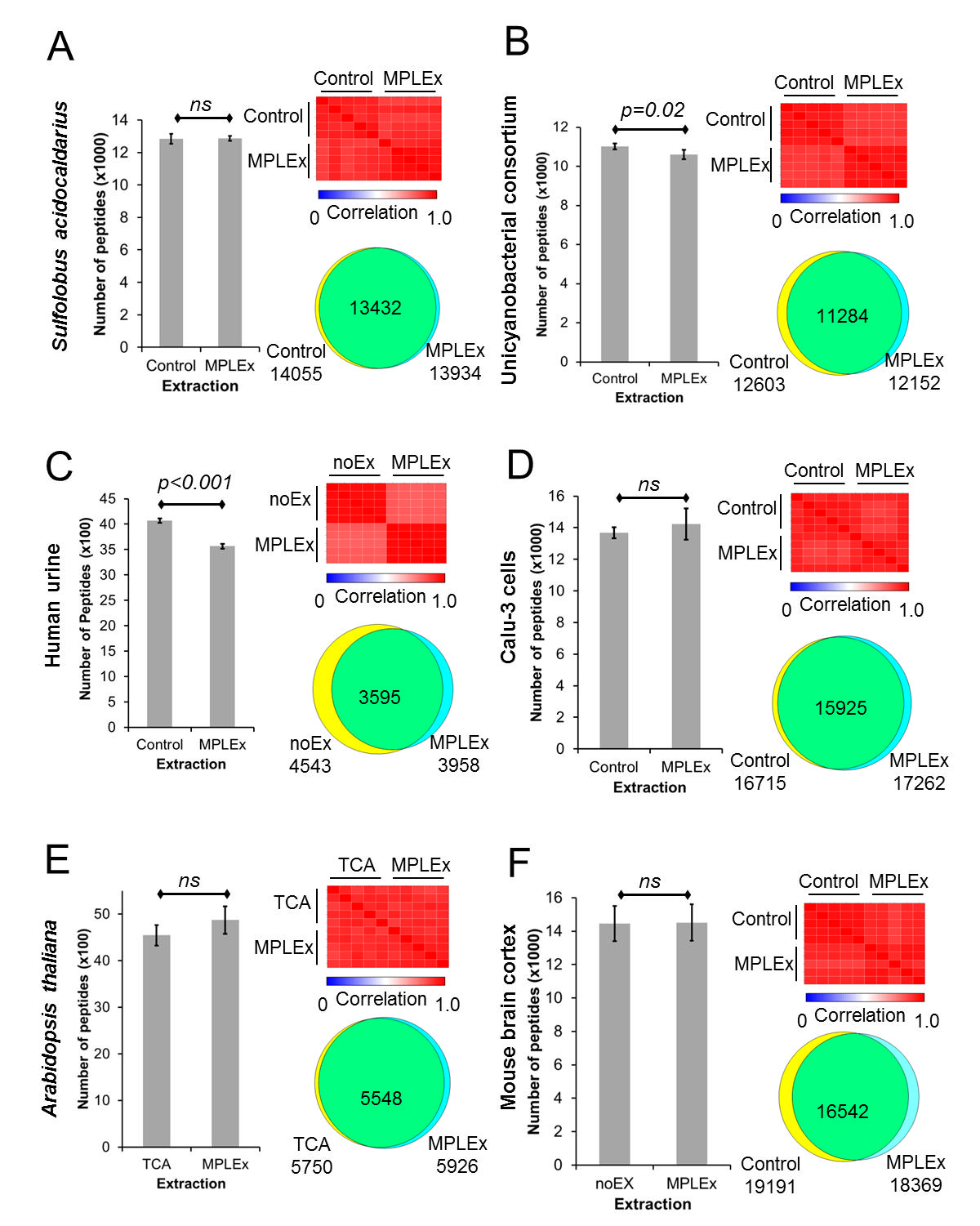

Supplement: Figure S1 [file sys003162021sf2.tif]

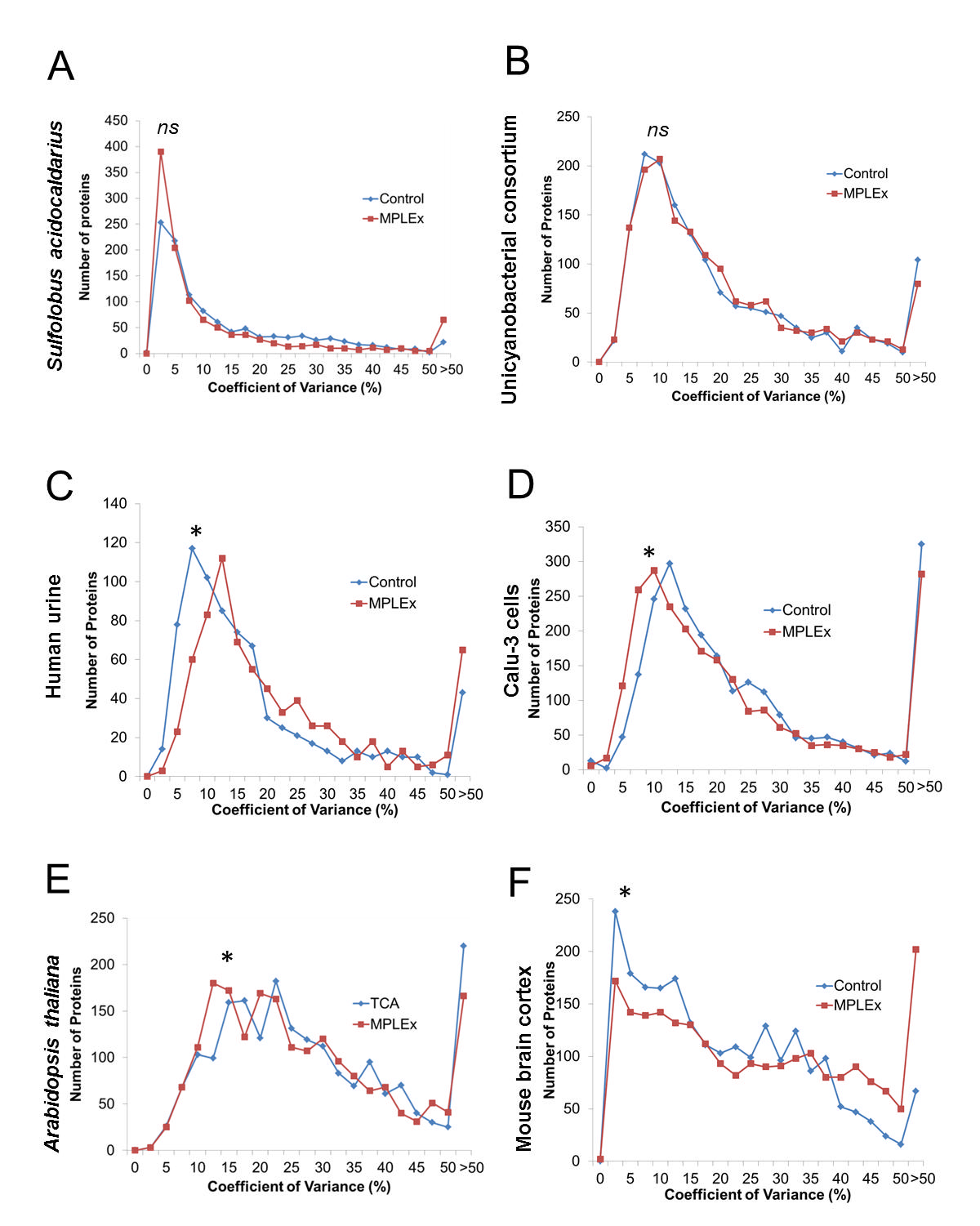

Supplement: Figure S2 [file sys003162021sf3.tif]

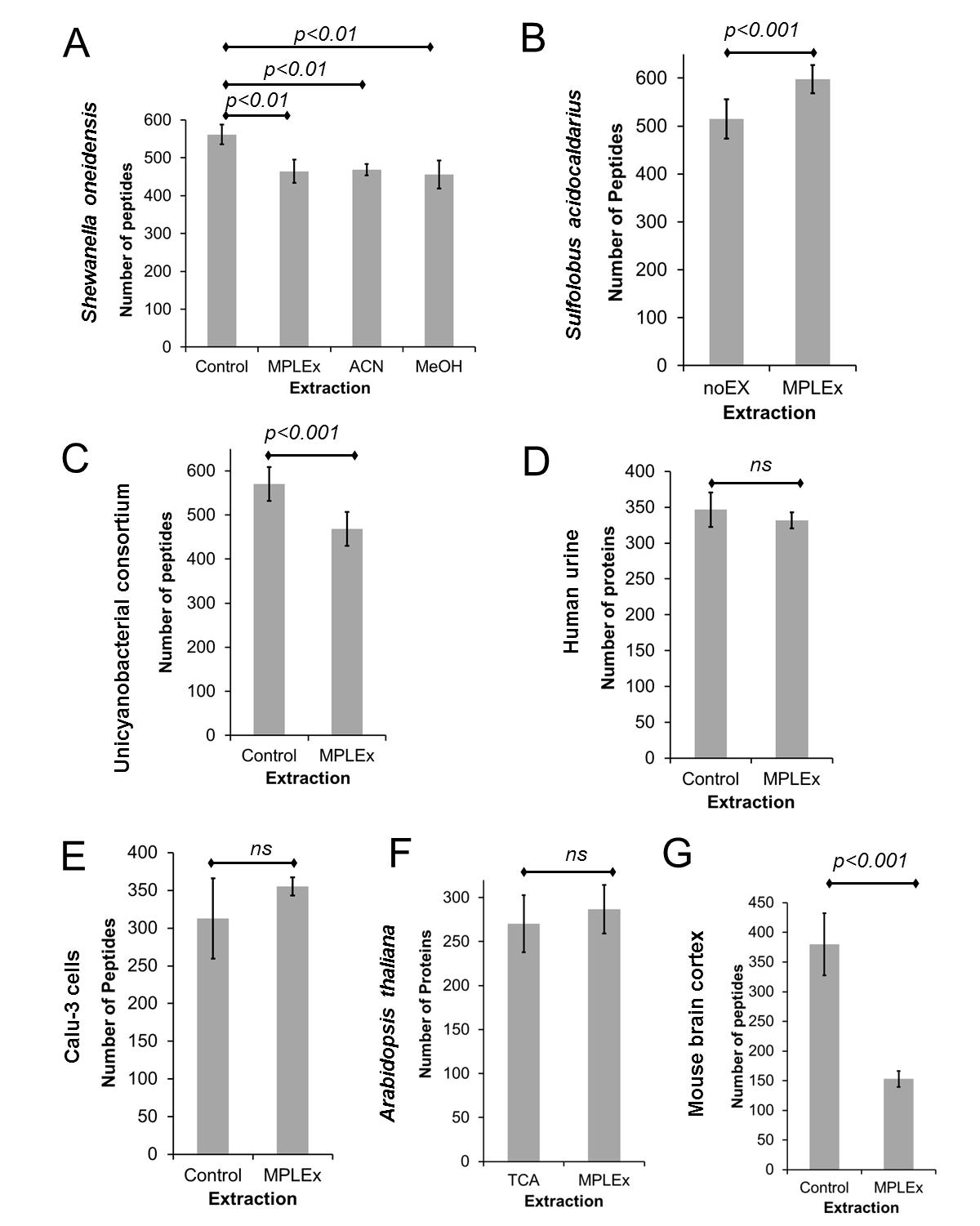

Supplement: Figure S3 [file sys003162021sf4.tif]
