# Supplementary material for: MPLEx: a Robust and Universal Protocol for Single-Sample Integrative Proteomic, Metabolomic, and Lipidomic Analyses
Source: mSystems. 2016 May 10;1(3):e00043-16. doi: 10.1128/mSystems.00043-16 (PMC5069757; doi:10.1128/mSystems.00043-16)
Supplement: Table S6 [file sys003162021st10.docx]

|  | **Sequence database** | | | | **Searching parameters** | | | | | |
| --- | --- | --- | --- | --- | --- | --- | --- | --- | --- | --- |
| **Sample** | **Species** | **Source** | **Date of download** | **# of entries** | **Tool** | **Enzyme digestion** | **Static Modification** | **Variable Modification** | **Parent mass torelance** | **Fragment mass torelance** |
| *Arapdopsis thaliana* | *Arapdopsis thaliana* | TAIR | Apr-02-2012 | 35,316 | MS-GF+ | Partial trypic | none | Methionine oxidation | 1.5 Da | optimized by MS-GF+ |
| Calu-3 cells | *Homo sapiens* | SwissProt/  Uniprot | Sep-18-2013 | 20,276 | MS-GF+/  DTArefinery | no enzymatic specificity | Cysteine carbamido-methylation | Methionine oxidation | 20 ppm | optimized by MS-GF+ |
| Unicyanobacterial consortium | *Microbial community* | Genome sequencing | Oct-04-2012 | 81,146 | MS-GF+/  DTArefinery | no enzymatic specificity | none | Methionine oxidation | 50 ppm | optimized by MS-GF+ |
| Human urine | *Homo sapiens* | SwissProt/  Uniprot | May-05-2010 | 20,276 | Sequest | no enzymatic specificity | Cysteine carbamido-methylation | Methionine oxidation | 3.0 Da | 0.8 Da |
| Mouse Cortex | *Mus musculus* | SwissProt/  Uniprot | Sep-18-2013 | 16,632 | MS-GF+/  DTArefinery | no enzymatic specificity | Cysteine carbamido-methylation | Methionine oxidation | 20 ppm | optimized by MS-GF+ |
| *Shewanella oneidensis* | *Shewanella oneidensis* | Genome sequencing | Apr-22-2010 | 4,300 | Xtandem | Partial trypic | none | Methionine oxidation | 3.0 Da | 0.8 Da |
| *Sulfolobus acidocaldarius* | *Sulfolobus acidocaldarius* | GenBank | Oct-10-2012 | 2,223 | MS-GF+ | Partial trypic | none | Methionine oxidation | 50 ppm | optimized by MS-GF+ |
