# Supplementary material for: MPLEx: a Robust and Universal Protocol for Single-Sample Integrative Proteomic, Metabolomic, and Lipidomic Analyses
Source: mSystems. 2016 May 10;1(3):e00043-16. doi: 10.1128/mSystems.00043-16 (PMC5069757; doi:10.1128/mSystems.00043-16)
Supplement: Text S1 [file sys003162021s1.docx]

**MPLEx: A robust and universal protocol for single sample integrative proteomic, metabolomic and lipidomic analyses**

Ernesto S. Nakayasu^1^, Carrie D. Nicora ^1^, Amy C. Sims^2^, Kristin E. Burnum-Johnson^1^, Young-Mo Kim^1^, Jennifer E. Kyle^1^, Melissa M. Matzke^1^, Anil K. Shukla^1^, Rosalie K. Chu^1^, Athena A. Schepmoes^1^, Jon M. Jacobs^1^, Ralph S. Baric^2,3^, Bobbie-Jo Webb-Robertson^1^, Richard D. Smith^1^, Thomas O. Metz^1^

^1^Biological Sciences Division, Pacific Northwest National Laboratory, Richland, WA, 99352 USA

^2^Department of Epidemiology, University of North Carolina at Chapel Hill, Chapel Hill, North Carolina, USA

^3^Department of Microbiology and Immunology, University of North Carolina at Chapel Hill, Chapel Hill, North Carolina, USA

**Supplemental Material and Methods**

**Middle East respiratory syndrome Coronavirus (MERS-CoV) infection**

Wild type infectious clone of MERS-CoV was obtained as previously described (1), and the viral genome was confirmed by sequence analyses prior to use in any experiments. Calu3 2B4 cells were plated 48 hours before infection, washed immediately prior to infection, infected with a multiplicity of infection of 5 (MOI 5) and incubated at 37 ^o^C for 40 minutes. The inoculum was then removed, cells were washed 3 times with PBS, and then fresh media added prior to time 0. At 18 hours post-infection, media was collected to determine viral titers and cells were washed once in cold Rapid Quench Buffer (150 mM ammonium bicarbonate and 60% methanol) and 150 µL of cold 150 mM ammonium bicarbonate buffer added to the cell monolayer which was then scraped and collected into a fresh 1.5mL siliconized microcentrifuge tube for extraction procedure. All work was performed in a biosafety level 3 facility supported by redundant fans, and samples were only removed from this facility after MPLEx extraction and testing for viable

**Proteomic analysis**

Proteomic analysis was performed using the accurate mass and time (AMT) tag approach (2). First, peptides were identified in extensive 2D-LC-MS/MS analyses using high pH reversed phase fractionation, as previously described in detail elsewhere (3), followed by LC-MS/MS analysis of each fraction using capillary reversed phase LC coupled with a linear ion trap alone (LTQ) or hybrid linear ion trap-orbitrap (LTQ-Orbitrap Velos; Thermo Fisher Scientific, San Jose, CA). Identified peptides are used to build a database containing their accurate masses and retention times, named mass tags, which are used to match and extract the peak areas of unfractionated peptides analyzed by subsequent 1D-LC-MS analyses using an Exactive or LTQ-Orbitrap mass spectrometer (Thermo Fisher Scientific).

Cell pellets or extracted proteins were dissolved in 100 mM NH_4_HCO_3_ containing 8 M urea and the protein concentration was measured by BCA assay. Disulfide bonds were reduced by adding dithiothreitol to a final concentration of 5 mM and incubating at 60 ºC for 30 min. Samples derived from mammals were alkylated with a final concentration of 40 mM iodoacetamide for 1 h at 37 ºC, while the samples derived from other species were not alkylated. The reaction was then diluted 10 fold with 100 mM NH_4_HCO_3_ followed by the addition of CaCl_2_ to 1 mM final concentration. Digestion was carried out for 3 h at 37 ºC with 1:50 (wt:wt) trypsin-to-protein ratio. Salts and reagents were removed by solid-phase extraction using C18 cartridges according to the manufacturer instructions and the resulting peptides were dried in a vacuum centrifuge.

Fractionated or unfractionated peptides were resuspended in milliQ water and 500 ng of material was loaded onto reversed-phase capillary columns (70 cm x 75 µm i.d. ; Polymicro Technologies Inc., Phoenix, AZ) packed in-lab with 3-µm Jupiter C18 stationary phase (Phenomenex, Torrence, CA)). The separation was carried out using a fully automated LC system, described in details elsewhere (4), using an exponential gradient of 0.1% formic acid in water (mobile phase A) and 0.1% formic acid in acetonitrile (mobile phase B) at 10 kpsi and ~500 nL/min. Alternatively, samples were analyzed in a 1200 nanoHPLC system (Agilent Technologies, Santa Clara, CA) by loading peptides into a trap column (5 cm x 150 µm i.d. column packed in-lab with 5-µm Jupiter C18 stationary phase) and separated in a 40 cm x 75 µm i.d. column packed in-lab with 3-µm Jupiter C18 stationary phase. The elution in this alternative chromatography was carried out at 300 nL/min with the following gradient: 0-8% B solvent in 2 min, 8-20% B in 18 min, 12-30% B 55 min, 30-45% B in 22 and 97-100% B in 3 min, before holding for 10 min at 100% B. Eluting peptides were introduced to the mass spectrometer using electrospray ionization with chemically etched fused silica emitters (5). Mass spectra were collected from 400-2000 *m/z* with 100k resolution at *m/z* 400 in both LTQ-Orbitrap Velos or Exactive mass spectrometers. Low resolution tandem-mass spectra were collected in the LTQ or LTQ-Orbitrap Velos by data-dependent acquisition of the ten most intense ions using normalized collision energy of 35%. A dynamic exclusion time of 60 s was used to discriminate against previously analyzed ions.

Mass tag databases were built by searching tandem mass spectra against species-specific sequence databases using Sequest (6), XTandem (7), MSGF+ (8) in combination or not with DTArefinery (9) to recalibrate the mass spectra. Details about the sequence databases and searching parameters are listed in **Table S6**. Mass tag databases were populated after filtering peptide-spectrum matches with a MS-GF score of 1e-10. Mass tags were then matched against the high resolution LC-MS runs and the peak areas were extracted using VIPER based on mass accuracy ≤10 ppm and NET ≤ 0.025 values (10). Matching results were filtered with Statistical Tools for AMT tag Confidence (STAC) using a score ≥ 0.7 and uniqueness probability ≥ 0.5. To improve the quality of the quantification, proteins were required to have at least 2 peptides and at least one peptide with STAC ≥ 0.9.

**Lipidomic analysis**

Lipids extracted from Calu-3 cells infected with MERS-Coronavirus were reconstituted in 50 µL of isopropanol and analyzed by LC-MS/MS as previously described (11). Samples were injected (0.7 µL) onto a capillary column (26 cm x 150 µm i.d.) packed with reversed-phase beads (1.8 µm, HSS T3, Waters, Milford, MA) and separated with a 90 min gradient (mobile phase A: acetonitrile/water (40:60) containing 10 mM ammonium acetate; mobile phase B: acetonitrile/isopropanol (10:90) containing 10 mM ammonium acetate) at a flow rate of 1 µL/min on a NanoAcquity UPLC (Waters).  Eluting molecules were directly analyzed in both positive and negative ionization in a LTQ-Orbitrap Velos and the top 6 most intense ions were fragmented by higher-energy collision dissociation (30% normalized collision energy) and collision-induced dissociation (35% normalized collision energy).  LC-MS/MS raw data files were imported into the in-house developed software LIQUID (Lipid Informed Quantitation and Identification) for semi-automated identification of lipid molecular species.  Confident lipid identifications were determined by examining the tandem mass spectra for diagnostic ion fragments along with associated chain fragment information.  In addition, the isotopic profile, extracted ion chromatogram (XIC), and mass error of measured precursor ions were examined to assist in identifications.

**Metabolomic analysis**

Polar metabolites extracted from Calu-3 cells infected with MERS-Coronavirus were derivatized as described previously (12). In short, 20 μL of methoxyamine in pyridine (30 mg/mL) were added to each sample, followed by incubation at 37°C with shaking for 90 min. Next, 80 μL of N-methyl-N-(trimethylsilyl)trifluoroacetamide (MSTFA) with 1% trimethylchlorosilane (TMCS) were added to each vial, followed by incubation at 37°C with shaking for 30 min. The samples were allowed to cool to room temperature and were then analyzed by GC-MS in a random order using an Agilent GC 7890A coupled with a single quadrupole MSD 5975C (Agilent Technologies, Inc, Santa Clara, CA). One microliter of sample was injected with the port in the splitless mode set at 250°C, and the separation was performed in a HP-5MS column (30 m × 0.25 mm × 0.25 μm; Agilent Technologies, Inc.) using the following gradient: temperature was held at 60°C for 1 min, and then increased to 325°C by 10°C/min, followed by holding for 5 min hold at 325°C.

GC-MS raw data files were processed using MetaboliteDetector (13). Briefly, chromatographic retention indices (RI) were calculated based on the elution profile of fatty acid methyl ester standards (C8–C28), then peaks were aligned and deconvoluted. Metabolites were then identified by matching GC-MS features, which consist of retention indices and deconvoluted mass spectra, against the Agilent Fiehn Metabolomics Library (14) with additional in-house determined metabolites, and all identifications were manually validated.

**References**

1. **Scobey T, Yount BL, Sims AC, Donaldson EF, Agnihothram SS, Menachery VD, Graham RL, Swanstrom J, Bove PF, Kim JD, Grego S, Randell SH, Baric RS.** 2013. Reverse genetics with a full-length infectious cDNA of the Middle East respiratory syndrome coronavirus. Proc Natl Acad Sci U S A **110:**16157-16162.

2. **Zimmer JS, Monroe ME, Qian WJ, Smith RD.** 2006. Advances in proteomics data analysis and display using an accurate mass and time tag approach. Mass Spectrom Rev **25:**450-482.

3. **Wang Y, Yang F, Gritsenko MA, Wang Y, Clauss T, Liu T, Shen Y, Monroe ME, Lopez-Ferrer D, Reno T, Moore RJ, Klemke RL, Camp DG, 2nd, Smith RD.** 2011. Reversed-phase chromatography with multiple fraction concatenation strategy for proteome profiling of human MCF10A cells. Proteomics **11:**2019-2026.

4. **Livesay EA, Tang K, Taylor BK, Buschbach MA, Hopkins DF, LaMarche BL, Zhao R, Shen Y, Orton DJ, Moore RJ, Kelly RT, Udseth HR, Smith RD.** 2008. Fully automated four-column capillary LC-MS system for maximizing throughput in proteomic analyses. Anal Chem **80:**294-302.

5. **Kelly RT, Page JS, Luo Q, Moore RJ, Orton DJ, Tang K, Smith RD.** 2006. Chemically etched open tubular and monolithic emitters for nanoelectrospray ionization mass spectrometry. Anal Chem **78:**7796-7801.

6. **Eng JE, McCormack AL, Yates JR, 3rd.** 1994. An approach to correlate tandem mass spectral data of peptides with amino acid sequences in a protein database. Journal of the American Society for Mass Spectrometry **5:**976–989.

7. **Craig R, Beavis RC.** 2004. TANDEM: matching proteins with tandem mass spectra. Bioinformatics **20:**1466-1467.

8. **Kim S, Pevzner PA.** 2014. MS-GF+ makes progress towards a universal database search tool for proteomics. Nat Commun **5:**5277.

9. **Petyuk VA, Mayampurath AM, Monroe ME, Polpitiya AD, Purvine SO, Anderson GA, Camp DG, 2nd, Smith RD.** 2010. DtaRefinery, a software tool for elimination of systematic errors from parent ion mass measurements in tandem mass spectra data sets. Mol Cell Proteomics **9:**486-496.

10. **Monroe ME, Tolic N, Jaitly N, Shaw JL, Adkins JN, Smith RD.** 2007. VIPER: an advanced software package to support high-throughput LC-MS peptide identification. Bioinformatics **23:**2021-2023.

11. **Gao X, Zhang Q, Meng D, Isaac G, Zhao R, Fillmore TL, Chu RK, Zhou J, Tang K, Hu Z, Moore RJ, Smith RD, Katze MG, Metz TO.** 2012. A reversed-phase capillary ultra-performance liquid chromatography-mass spectrometry (UPLC-MS) method for comprehensive top-down/bottom-up lipid profiling. Anal Bioanal Chem **402:**2923-2933.

12. **Kim YM, Schmidt BJ, Kidwai AS, Jones MB, Deatherage Kaiser BL, Brewer HM, Mitchell HD, Palsson BO, McDermott JE, Heffron F, Smith RD, Peterson SN, Ansong C, Hyduke DR, Metz TO, Adkins JN.** 2013. Salmonella modulates metabolism during growth under conditions that induce expression of virulence genes. Mol Biosyst **9:**1522-1534.

13. **Hiller K, Hangebrauk J, Jager C, Spura J, Schreiber K, Schomburg D.** 2009. MetaboliteDetector: comprehensive analysis tool for targeted and nontargeted GC/MS based metabolome analysis. Anal Chem **81:**3429-3439.

14. **Kind T, Wohlgemuth G, Lee do Y, Lu Y, Palazoglu M, Shahbaz S, Fiehn O.** 2009. FiehnLib: mass spectral and retention index libraries for metabolomics based on quadrupole and time-of-flight gas chromatography/mass spectrometry. Anal Chem **81:**10038-10048.
